# A qualitative interview study of patients' attitudes towards and intention to use digital interventions for depressive disorders on prescription

**DOI:** 10.3389/fdgth.2024.1275569

**Published:** 2024-02-05

**Authors:** Jacqueline Posselt, Eva Baumann, Marie-Luise Dierks

**Affiliations:** ^1^ Institute for Epidemiology, Social Medicine and Health Systems Research, Hannover Medical School, Hannover, Germany; ^2^ Department of Journalism and Communication Research, Hanover University of Music, Drama and Media, Hannover, Germany

**Keywords:** acceptance, digital interventions, depressive disorders, qualitative research, mental health, family medicine

## Abstract

**Background:**

Depressive disorders are an emerging public health topic. Due to their increasing prevalence, patients with depressive disorders suffer from the lack of therapeutic treatment. Digital health interventions may offer an opportunity to bridge waiting times, supplement, or even substitute in-person treatment. Among others, the Unified Theory of Acceptance and Use of Technology (UTAUT) explains that actual technology use is affected by users' behavioural intention. However, patients' perspectives on digital interventions are rarely discussed within the specific context of primary care provided by general practitioners (GP) and need further exploration.

**Method:**

A qualitative study design with semi-structured interviews was used to explore DTx-acceptance of patients with mild or moderate depression (*n* = 17). The audio-recorded interviews were transcribed verbatim, coded, and thematically analysed by qualitative content analysis.

**Results:**

Patients' *performance expectancies* reveal that DTx are not perceived as a substitute for face-to-face treatment. *Effort expectancies* include potential advantages and efforts concerning technical, motivational, and skill-based aspects. Moreover, we identified *health status* and *experience with depressive disorders* as other determinants and potential barriers to patients' DTx acceptance: Difficult stages of depression or long-time experience are perceived hurdles for DTx use. *GPs'* recommendations were just partly relevant for patients and varied according to patients' consultancy preferences. But still, GPs have a crucial role for access due to prescription. GPs' influence on patients' DTx acceptance varies between three situations: (1) pre-use for consultation, (2) pre-use for access and (3) during DTx-use. Further, GPs' guidance could be especially relevant for patients during DTx-use in routine care.

**Discussion:**

The UTAUT-based exploration suggests that acceptance determinants should be considered independently and embedded in personal and situational aspects. DTx require a healthcare professional to prescribe or diagnose the disease, unlike other digital offerings. We identified prescription- and depression-related determinants, exceeding existing theoretical constructs. GPs' guidance can compensate for some barriers to DTx use e.g., by increasing commitment and motivational support to strengthen patients' acceptance.

**Conclusion:**

We argue for a multidimensional integration of acceptance determinants for further development of health technology acceptance research. Future research should specify how DTx can be integrated into routine care to strengthen user acceptance.

## 1 Introduction

### 1.1 Depressive disorders and the burden of disease

Depressive disorders are one of the leading contributors to the burden of disease worldwide and among the most prevalent mental illnesses in Germany (1, 2). People with depression often struggle with access to mental health care and waiting times for specialist or psychotherapeutic treatment (3–6). Especially lower socio-economic groups are underserved by outpatient psychotherapy (7, 8). Accordingly, in the German health system, general practitioners (GPs) are the first contact for health concerns for many patients with depressive disorders and essential providers of basic psychosomatic care (9, 10). Given the lack in mental health care supply, patients do not always receive treatment according to guideline recommendations (11–13). Hence, prevention and healthcare provision for depressive disorders are important subjects and tasks for public health (4, 14).

An innovative approach in mental health care that might contribute to reducing this lack of therapeutic treatment are internet-based, digital interventions (4, 15–17). The number and variety of digital health services for mental disorders is constantly increasing over the last decade (18). Digital health services for mental health include health promotion, prevention, or treatment of some disorders, e.g., by providing behavioural information to encourage patients’ self-management (19, 20). Digital interventions for mental health can be applied with professional guidance to assist and follow up the use, completely self-guided by patients or as blended approaches as an additional part of face-to-face treatments (13, 16).

In 2020, Germany was the first country worldwide where specific software applications (so-called Digital Therapeutics (DTx) for “Digitale Gesundheitsanwendungen” (DiGA)) became part of the German statutory health insurance services (21). DTx are defined as digital, low-risk medical devices to identify, monitor, treat or compensate for illnesses or disabilities (§33a SGB V). General characteristics include that they are for a specific medical condition such as depression, contain a therapeutic intervention, and are considered approved medical applications by regulatory bodies (22). To become temporarily or permanently listed in a DTx catalogue, digital applications have to pass a review process by the Federal Institute for Drugs and Medical Devices (BfArM) (23). Listed DTx have proven a positive medical benefit (e.g., improve health status, reduce disease duration, improve quality of life) or patient-relevant process improvements such as increased coordination, guideline treatment or adherence (24). DTx are available on prescription, patients can receive them from physicians or therapists or directly from statutory health insurance companies. Costs for available mental health DTx are covered by statutory health insurance vary between 178,50 € and 855,82 € per quarter (21). Almost 90 percent of patients in Germany across all disorders receive these prescriptions from physicians, mainly prescribed by GPs (25). Still, the recommendation of digital health interventions for people with depression or prescription of DTx in Germany is limited (26–28). Recently Löbner et al. (2022) identified simply forgetting and a shortage of time as major reasons for the little uptake of digital interventions by GPs in routine care. Users' acceptance is also a precondition for implementing digital mental programmes in routine care (29, 30), but little is known about patients' acceptance of DTx.

### 1.2 Acceptance of digital health interventions

The intention to use certain technologies and actual use is explained by technology acceptance models such as the Technology Acceptance Model (TAM) (Davis 1989) or the Unified Theory of Acceptance and Use of Technology (UTAUT) (31, 32). The UTAUT is a consolidated model, combining behavioural intention and technology acceptance models (31). According to UTAUT, the intention to use technology and actual use depends on four determinants: Performance expectancy, effort expectancy, social influence and facilitating conditions, moderated by age, gender, experience and voluntariness of an individuals' use (31). Moreover, the UTAUT is used in various research disciplines and can be successfully adapted for the health sector (33, 34), but it also needs adjustments according to the specific healthcare setting (35). Thus, the theory offers a solid framework for our research interest. At the same time, there is a risk that a strong alignment with a model may contribute to its reproduction, especially in acceptance research which nearly reached a plateau (36). This means research on technology implementation has to take health systems’ complexity into account. Currently, researchers use a variety of constructs to measure technology acceptance, which shows the need for a common approach in the specific domain of mental health (37, 38). According to our research interest, an explorative approach is needed to understand users' acceptance of DTx and indicate potentially different patient-specific influences on technology acceptance within a GP setting.

Previous research on health technology acceptance focuses on the perspective of stakeholders and health professionals, showing an ambivalent acceptance of professions towards digital interventions for mental health (29, 39, 40). Moreover, in Germany GPs' acceptance of DTx seems greater compared to regular health apps (41). Patients' acceptance of health technologies in general is less studied (34), such as specifically the acceptance of digital interventions for mental health. Further, many research results generally do not sufficiently distinguish between different digital approaches in mental health or DTx and non-medical applications (42, 43). First results indicated that patients' acceptance of digital interventions for depressive disorders in Germany in general seems limited (13, 44). Factors that promote patient engagement with digital mental health interventions are rarely applied within technology acceptance models (45). Further subgroups have to be understood to achieve patient-orientation within DTx implementation (24). Hence, it is unclear whether (GPs') prescription for digital interventions impacts patients’ acceptance or rejection (46). Further, it is unknown from patients' perspectives which factors affect acceptance of prescribed digital interventions.To explore patients' acceptance of DTx, we defined performance expectancies, effort expectancies and GPs' influence on DTx as determinants of DTx acceptance in accordance with UTAUT (*documented in* Table 1). *Performance expectancy* is defined as the degree to which patients believe that DTx will be helpful for their depressive disorder. *Effort expectancy* includes the degree to which patients believe that DTx are associated with ease of use. Primary medicine provided by GPs is often characterised by a continuous, long-standing relationship between patient and GP, a low-threshold consultation and GPs' knowledge of the psychosocial environment of their patients (47). Within a family medicine setting, GPs are both the institutional and personal point of contact for health issues and include aspects of social influence and facilitating conditions. Therefore, *GPs' influence* is defined as patients' interest in DTx-recommendation by GPs and the degree to which patients believe their GP is supportive of DTx-care. We chose UTAUT because it is based on the key determinants for our research objective, while its extension (UTAUT II) includes other factors, such as cost, that are less relevant in the context of our question.

**Table 1:** UTAUT-Determinants according to the research questions.

UTAUT-Determinants	Determinants according to the research questions	Definition for this research
	Patients’ experience	Patients’ experience include patients’ descriptions of their previous experiences with digital services in the context of depression.
Performance Expectancy	Performance expectancy on DTx	Performance expectancy is defined as the degree to which patients believe that DTx will be helpful for their depressive disorder.
		This category includes expectancies on potential barriers and facilitators, e.g., health condition, disease management or access to care.
Effort Expectancy	Effort expectancy on DTx	Effort expectancy is defined as the degree to which patients believe that DTx are associated with ease of use.
		This category includes expectancies on potential barriers and facilitators, e.g., literacies or design.
Social Influence	GPs’ Influence	GPs’ influence contains: 1) The perceived meaning of a GPs’ DTx-recommendation for patients 2) The degree to which a patient believes his or her GP is supportive of DTx-use (e.g., with knowledge, access, or competence) This category includes expectancies on potential barriers and facilitators.
Facilitating Conditions		
Behavioural Intention	Behavioural Intention	Behavioural intention is defined as patients’ intention to use a DTx for depressive disorders.

We aimed to contribute to the further development of acceptance research by exploring patients' perspectives on DTx within a primary GP medicine with the following questions:

1) What are performance and effort expectancies encouraging or discouraging patients from the (intention to) use DTx?
2) What role do GPs play regarding the intention to use DTx from a patient's perspective?
3) What are patients' intentions to use digital interventions for depressive disorders?

## 2 Materials and methods

### 2.1 Research design

Little is known so far about patients' expectancies, intention to use and actual use of DTx for depressive disorders. Beyond technology features end-user perceptions and characteristics must be considered to achieve user-centred technologies (48). A qualitative interview design was chosen to achieve an in-depth focus on potential users, strengthening a patient-centered perspective on DTx in routine care. Therefore, we were interested in capturing patients' perspectives and experiences in everyday life, disease-specific aspects according to DTx acceptance, and particularities that may result from being prescribed by a physician. Consolidated Criteria for Reporting Qualitative Research (COREQ, Appendix 1) were consulted to comply with quality standards in research.

### 2.2 Sampling and recruiting

We aimed to achieve a heterogeneous sample of patients with mild or moderate depressive disorders without current specialist or psychotherapeutic treatment in primary care provided by a GP. All participants had to be above the age of 18. Due to purposeful sampling, we composed variation in terms of sex, geographic distribution, current perceived health status, and disease biography [*Categories of the sampling process are documented in* Table 2]. Participants were recruited with posters and flyers in GP offices, medical counselling centres, and self-help facilities for people with depressive disorders. The call for participation contained the topic of the survey and information about the estimated duration of the interview. It introduced the research as part of the doctoral programme “Chronic Diseases and Health Literacy”.

**Table 2:** Characteristics of participants.

Sex	Male	6
	Female	11
Education	Middle-School degree	2
	Professional training	9
	Higher education (A-Level and university degree)	6
Depression duration	Less 1 year	3
	1–5 years	3
	Above 10 years	11

### 2.3 Data collection

The inclusion criteria were checked before the start of the interview based on participants' self-report. We used a semi-structured interview guide, following the main topics (A) Experience with digital health interventions for depressive disorders, (B) Potential chances, risks, and barriers of DTx, (C) DTx within a primary medicine setting provided by GPs and (D) Participants' intention to use DTx. At the time of the interviews, less than 20 DTx were available, therefore a short video was used as stimuli material to exemplify DTx interventions for patients. In the first part of the video, we defined DTx and explained how they differ from other apps and showed the process for patients to receive them. Further, we showed the participants examples of DTx for patients with depressive disorders. These examples included product descriptions from the DTx-catalogue and images of registered DTx to illustrate topics, structure, and use of the tool. The authors developed the interview questions and pretested them by two volunteers: A patient with a chronic disease and a patient with major depression. To explore user-centered topics, open-ended questions were conducted in the interview guide. Participants could freely describe their experiences and attitudes, further questioning was used to deepen aspects relevant for the research. All interviews were carried out between January and June 2022 by the first author in German. They were conducted via video meetings, data were audio recorded, transcribed verbatim, and anonymised according to Dresing and Pehl (49). All participants gave verbal consent prior to the start of the interviews. Anonymity and confidentiality were maintained. Participants also declared informed consent for the audio recording and scientific use of the interviews by written consent form and received reimbursement (15 €) for participation. The interviewees were not known prior to the interview. Field notes were taken during interviews to document non-verbal elements e.g., interruptions. Recorded interviews lasted between 24 and 42 min. The study has received ethical approval from the Ethics Committee of Hanover Medical University (No. 10131_BO_K_2021).

### 2.4 Data analysis

**Figure 1:**
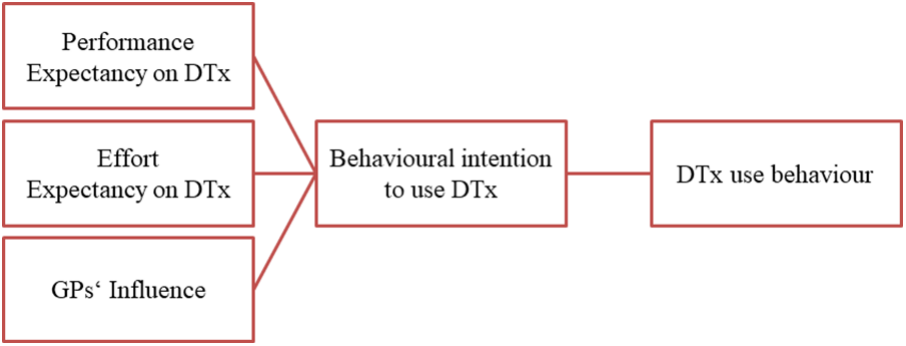
UTAUT determinants according to the research question.

According to Kuckartz and Rädiker (50), a qualitative content analysis was carried out to analyse the material. A coding scheme was built deductively along the interview guide and UTAUT-determinants and expanded within the main categories inductively during the coding process. For this purpose, all transcripts were first coded to build the coding scheme and afterwards to apply the material along the scheme. A second researcher coded the interview material (25%) independently, conflicts in coding were compared and discussed within consensus coding to ensure the unambiguity of the categories. The results are reported according to the research questions, which are based on the UTAUT-determinants *(see also* Figure 1):

• Patients' performance expectancy
• Patients' effort expectancy
• GPs' influence
• Behavioural intention to use DTx

According to the openness of qualitative research, we interpreted the acceptance determinants with an equal impact. To technically support the coding and analysis process the software MAXQDA (2022) was used. The research project was regularly presented in a qualitative research workshop where both coders participated to discuss and reflect on the procedure and interpretation of results. Relevant quotes from interview sequences were translated from German into English.

## 3 Results

### 3.1 General characteristics of study participants

A total of 17 patients with mild or moderate depressive disorders participated (*demographics are shown in* Table 2). Eleven participants were female, eleven patients reported a depression history of above ten years, and three patients suffered from depression for less than one year. Nine participants completed professional training, while six patients had a higher education by completing an A-level or a university degree. Ten patients lived in urban or suburban areas.

Based on their experience with (prescribed) digital interventions, the sample is divided into four different user groups: Eight participants hadn't heard about DTx before the interview. Six participants who heard about it before but did not plan to use it or looked for further information, and one patient heard about it and was informed to propose it to a health professional. In the fourth group, patients had no experience with DTx but already used various other depression-specific digital services, from mood diaries to telemedicine consultations or meditation apps.

### 3.2 Patients' performance expectancy on DTx

Performance expectancy comprises patients' estimation on how DTx might achieve changes for the current healthcare situation. Participants reported on performance expectancies on DTx, but also compared DTx with in-person psychotherapy.

According to the participants (*n* = 15), DTx have a meaningful potential to bridge waiting times until patients receive appointments for further in-person treatments. During waiting times for psychological or specialist treatment, participants see a chance to increase their self-management skills. Additional support and accompaniment for “*new impulses*” (P13:48) to “*get out of the problem yourself and perhaps deal with it in a slightly different way*” (P18:29). One participant even considered DTx as a substitute for pharmaceuticals during waiting times.

Participants who were presently in good condition, described DTx as a fall-back option for difficult mental health situations in the future:

*“Because imagine you’re in a depressed situation and then you know in the back of your mind that you might have to wait another eight months for a treatment.” (P14:48)*

Some patients emphasise the emotional burden of waiting for therapeutical treatment and consider the knowledge about available alternative options as essential “*even if it's just mentally, to know that you have a chance.”* (P3:50). In this context, patients see further advantages as motivational support to improve coping with depressive disorders.

In contrast to general health information from the internet, seven participants experience information provided by DTx as more trustworthy and feel more secure using them and expect access to evidence-based information:

*“Yes, this informative, so that you can simply read something about yourself. An information that is not somehow from Google, but medically correct.” (P2:45)*

Further participants expect improvements on access to health care professionals' advice especially for difficult situations due to communication tools such as chat within DTx:

*“So that I don’t feel completely helpless and alone about it. And still, get professional help somewhere.” (P6:45)*

Moreover, five participants expect greater anonymity as another essential advantage of digital interventions in mental health to avoid stigmatisation. While one part of the participants shares their experiences in depressive disorders with their social environment, the other part is convinced that mental health is a topic *„you don't peddle”* (P17:78)*.* According to the participants, admitting a mental disorder is challenging, so a tool could reduce this burden without letting friends or family know.

*“That's the very point why I think the app is good: Many people can’t even tell their best friends, siblings, or parents how they’re feeling. So why tell a therapist all of a sudden?” (P3:58)*.

Apart from the performance expectations that relate to the DTx itself, participants describe disease- and healthcare-related influences that may limit performance expectations: Even though the participants expect advantages compared to unguided waiting times in routine care, none of them perceive digital health interventions as an alternative or substitute for psychotherapy or face-to-face consultation. Generally, DTx are considered to be less effective in improving health conditions than treatment in-person:

*“Although I don’t think I would see this as a substitute for a real conversation with a psychologist. But to bridge the time until you have a therapy place, I could imagine that it would be helpful.” (P9:33)*.

According to some expectations, digital treatment options include a high level of standardisation. The participants perceived that depressive disorders are too complex and unique for a total standardised treatment option (*n* = 5):

*“It is not a purposeful alternative because the psyche of everyone is simply too individual for that.” (P18:41)*.

Besides perceived positive performance aspects, patients also have certain essential worries that could already deny the actual use or effect of those offers. According to the participants, a central barrier is the interplay between the disease and digital technologies (*n* = 13). Digital media are perceived as potential triggers or amplifiers for mental health issues and depression. The idea of such situations without personal and therapeutic support is a concern of the participants.

*“If you know that there's a robot, I’m having a panic attack or whatever, because some thoughts are just coming up, that would be my worry, I’d say.” (P10:62)*

Moreover, patients expect further adverse effects on their health, e.g., by too frequent use of technology or “*too much brooding” (P15:32)*.

Another reason not to take further interest in the use of technology is the underlying therapeutic approach (*n* = 4). Currently, many DTx for mental diseases are based on cognitive behavioural therapy (21). Some of the more experienced patients assessed this therapy approach as not the right treatment option for themselves based on their therapeutical history:

*“No. I wouldn't use it because, based on my therapeutic experience, my priority is the relationship with the person. And no app can learn that. I need a person for that.” (P7:61)*

Further, performance expectancies include a variety of advantages of DTx such as tools to improve self-management skills and low-threshold access to approved health information for a limited period. Overall, participants expect lower efficacy of DTx compared to face-to-face therapy. Also, worries on opposing effects limit patients' performance expectancy on DTx.

### 3.3 Patients' effort expectancy on DTx

Patients' performance expectancy is the degree of ease associated with using DTx for depressive disorder. Moreover, performance expectancy includes learning and operating DTx.

Participants await more flexibility using health technologies as a “*low-threshold offer” (P16: 48)* according to personal needs (*n* = 9). Especially experienced patients who used different digital interventions for mental health reported less effort in contrast to regular mental health supply (*n* = 11). Access to digital health intervention seems less time and energy-consuming in a lethargic period.

*“Because these waiting times really drain your energy, and you don’t feel like calling the fifth therapist who doesn’t have an appointment for you. And this back and forth, and yes, that makes this time, I don’t know this app yet, but probably more bearable.” (P10:60)*

Overall, additional efforts were rarely mentioned by the participants for themselves. Patients perceived little efforts or challenging factors for specific groups e.g., with less access to digital technologies or infrastructure:

*“I was thinking more of (…) older people who are perhaps not that fit and perhaps don’t necessarily have a mobile phone or don’t have a computer or a tablet. That's really not a problem for me, but it might be for other people.” (P18:51)*

In addition, digital skills could cause effort for less experienced users to become familiar with DTx (*n* = 12). Moreover, all participants depicted a challenge to regularly use DTx independently. Personal appointments in the therapy setting are perceived to be more compulsory.

In summary, *effort expectancies* on DTx revealed ease of access to mental health care and greater convenience due to digital opportunities. Expectancies include also potential barriers for people with little digital skills or competencies to become familiar with DTx.

### 3.4 GPs' influence on acceptance

Due to our result, *GPs' influence* on DTx acceptance varies between three situations: for consultation, for access and after prescription in routine care.

The participants reported two different positions towards GPs' consultation: Participants who experienced a trustful relationship with their GPs, assessed GPs' recommendations as highly relevant or even essential for decisions on DTx-use.

*“But I also ask the doctor how her experiences with it are so far. For example, whether she [the doctor] has a [DTx-] provider with whom she has had very good experiences with her patients so far. That would be important to me because what she says is very, very important.” (P17:102)*

In contrast, participants complained about the insufficient knowledge of GPs on digital interventions and mental health as marginal topics of GP training, qualification, and practice which limits the expectations on GP's recommendations (*n* = 9). Especially participants with experience on digital mental health interventions, experienced in single cases insufficient empathy from their GP which discourages them from addressing mental health issues.

*“I don't really talk to my GP. He's a very strict traditional doctor. You go there when you have a cold, that's it.” (P5:65)*

Thus, patients' relevance on DTx-recommendations from GPs varies, they are overall seen as important gatekeepers regardless from participants' personal attitude towards DTx (*n* = 9). GPs were described as necessary for prescriptions as patients rarely get to know the offers by themselves:

*“Well, I think that doctors should also offer this because you don't learn anything about it.” (P4:70)*

Even though DTx could be prescribed by different health professionals and received from health insurances directly, GPs are perceived as low-threshold prescribers. Therefore, GPs are seen as necessary for a prescription even though patients do not claim mandatory consultancy.

*“I need the prescription. But I don't consult with her [the GP].” (P18:60)*

However, patients also raise concerns about GPs not fulfilling this function. GPs being unfamiliar with digital interventions could be a hurdle for DTx recommendation or prescription in primary care.

*“When I talk to my GP, it's always about sick notes, medication, rehabilitation, and so on. It's not about health apps.” (P8:68)*

Moreover, participants being critical of the empathy and competence of their GPs on mental disorders, estimate difficulties in receiving a prescription by their GP.

*“I could imagine this hurdle, I’m going to my doctor now, he might not know anything about it if you have mild depression or moderate.” (P5:69)*

Further, some participants liked to know earlier about the services to contact their doctor to raise awareness and receive a prescription (*n* = 8).

*“I think if I had already known about the app, I think I would have really, probably really approached my doctor and asked, can you please prescribe me something like that? Because I think just out of interest or this desire to help myself.” (P14:64)*

To sum up, GP influence manifests itself in different facets: On the one hand, as a perceived structural prerequisite to gain access and, on the other hand, as substantive advice, which in turn depends on the competence and experience of the GP. Patients' consultancy preference causes GPs' influence on patients' intention to use, but still, GPs seemed relevant to receive prescriptions for the actual use.

### 3.5 Intention to use DTx for depressive disorders

Overall, after the presentation of the services, ten participants expressed interest towards DTx for depressive disorders. Moreover, some patients could imagine using the services or planning to introduce those services to their doctor.

*“I found it very trustworthy. I would also make use of it.” (P6:36)*

The participants in our sample believed that the intention to use varied within the course of the disease. They assumed retrospectively that DTx would fit best to their needs in the early stage of depression and less experience with or knowledge about the disease (*n* = 11).

*“When you realise that you have to go, but just can’t go yet, but still work on yourself, that maybe you have a little, yes, a little support.” (P4:47)*

Further, participants claim that the actual health condition restricts the intention to use. Participants tend to see no need for DTx-use in good health, while in “*highly depressive phases where you’re not capable of anything” (P14:50),* the intention could be limited (*n* = 12).

Four Participants who declared no interest in the use explain this with their long-time experience with mental health care and depression by saying they don't see an extra value for themselves through the technology.

*“No, because of all the groundwork that the app would do, I've already done quite well in the year of therapy. And now it's just sitting on your arse and maintaining the level.” (P4:61)*

In summary, the intention to use DTx is unstable and varies depending on the disease stage, knowledge of depressive disorders and personal coping strategies. Further, health conditions and experience can have a negative impact on DTx use intention, detached from general performance and effort expectancies.

## 4 Discussion

### 4.1 Summary of main findings

**Figure 2:**
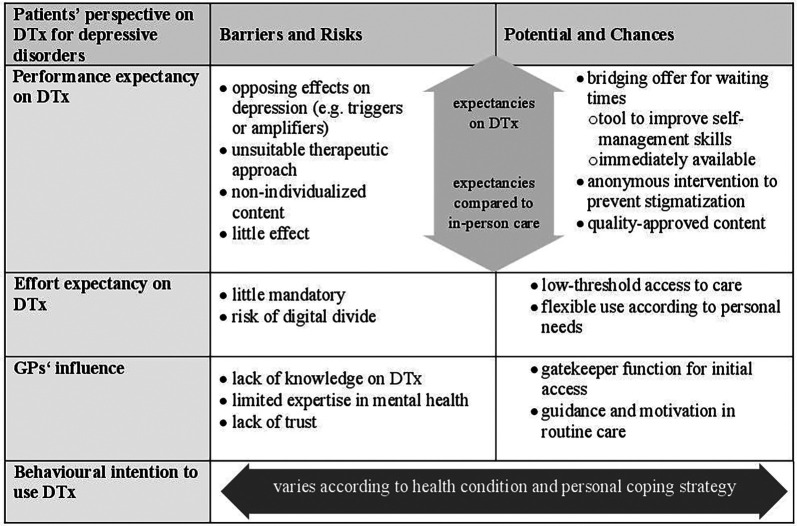
Summary of the main findings.

We aimed to explore inhibiting and promoting factors on patients' acceptance of prescribed digital health interventions in GP practice (Figure 2). Our research focused on patients' performance and effort expectancies and GPs' influence on the intention to use DTx for depressive disorders during waiting times. We identified expected chances and barriers on the determinants, leading to acceptance and non-acceptance on DTx. Further, we identified structural- and depression-related influences, affecting and exceeding single constructs. In conclusion, patients’ acceptance includes expectancies on DTx in a GP setting and compromises determinants such as the availability of treatment opportunities, health status and personal coping strategies.

### 4.2 Comparison with existing literature

This study examines patients in Germany according to their attitude towards digital mental health interventions. Although Germany appears to be a pioneer in the implementation of DTx, this innovativeness may not apply to the general population: In comparison to OECD countries, German citizens are for example less likely to seek health information on the internet (51). Previous research also shows that Germans show rather low acceptance rates toward the innovative mental health treatment forms (44). The results of our study help to understand the reasons for the limited acceptance from the patient's point of view.

#### 4.2.1 Expectancies on performance and effort

Thus far, the absence of timely face-to-face mental health care and current waiting times appear to be relevant context factors for patients to consider DTx for depressive disorders. Also Watanabe-Galloway et al. (2021) identified currently underserved patients with depression open-minded towards digital disease-specific interventions (52).

Due to the non-availability of in-person services, self-management skills become more important for patients as users of digital mental health care (20, 53). From patients' perspectives, our result showed that DTx could be a tool to improve coping with major depression for a period. Further, the need for access to approved health information, especially at an early stage of disease was presented. Participants perceive DTx a reliable source of information, but apart from that appraise them as less effective than face-to-face psychotherapy. Even though DTx are not perceived as a substitute, research shows comparable effects of digital interventions and face-to-face treatment (20, 54), but reliable evaluations for digital mental health interventions are not comprehensive (20, 55). According to the admission process, permanently listed DTx fulfilled higher evaluation standards (24) to examine effectiveness and contribute to evidence-based mental health care. Our findings suggest that knowledge about the effectiveness of digital mental health in patient care needs to be strengthened to increase patients' acceptance.

Former research identified digital literacy as a predictor for patients' acceptance of digital health interventions (46) and a potential barrier to professionals’ acceptance (56). Further digital technologies could reproduce social inequalities due to insufficient digital literacy, known as the digital divide (57, 58). In contrast to earlier findings, computer-specific literacies such as digital literacy or data security were little discussed and not identified as challenging for the participants themselves in our sample. Hence, these results must be interpreted cautiously because patients tend to overestimate their technology skills within digital health technologies (59). Also, self-reported competence and actual behaviour may differ in practical situations. Another interpretation of this finding could be that digital skills have a minor role for participants: Previous research on patients' mobile health adoption identified users' self-efficacy as an essential component for digital mental health interventions adoption (19, 46, 60, 61). Taken together, this suggests that patients tend to perceive digital skills as a small hurdle if they are convinced they may benefit from DTx.

Contrasting the digital divide, the study suggests that anonymity and impersonal access to health care could be advantages of DTx. This feature could be suitable for unprovided patients who fear in-person psychotherapy (6). Currently, attitudinal barriers (e.g., being afraid to disclose in front of others or people who fear stigmatisation) are the most common reasons to refuse to find help (5, 62). Under this assumption, DTx could be an alternative and low-threshold access for specific and underprovided groups in routine care.

#### 4.2.2 Implications for technology acceptance for mental health

Further, we identified *health status* and *experience with depressive disorders* as additional determinants on patients' acceptance of digital health interventions on prescription. Based on participants' previous illness biographies, the present study highlighted the challenges of depressive symptoms. Patients' intention to use digital health interventions is affected by their health status, which can be negatively influenced by difficult periods. Earlier qualitative research also explored potential opposing effects between depressive disorder and the use of technology in acute or critical situations (63). These effects might limit the DTx adoption in routine care, hence low user engagement compared to study conditions is a central challenge (45). According to Nadal et al. (2020), mobile health technology acceptance is a stage in a dynamic staged process. They distinguish between pre-use, initial use and post-adoption as different stages influencing technology acceptance (37). Our results indicate that acceptance research on (mobile) technologies for long-term disease also should take factors depending on the stage of disease into account. Currently, the state of the art in terms of acceptance research comes also to a limit regarding further disease-specific challenges such as access to treatment, stigma, symptoms, and dysfunctional effects. Therefore, health status and experience with depressive disorders are other determinants of patients' intention to use DTx, influencing different acceptance dimensions. Also, determinants of patients' acceptance are embedded in personal and situational aspects. Accordingly, acceptance determinants should not be considered independently, as correlation-based models such as UTAUT suggest. Further, the results imply a connection between DTx assessment, intention to use and action to achieve access by introducing DTx to a GP. Therefore, a multidimensional integration of acceptance determinants is needed.

#### 4.2.3 DTx in routine care

Unlike other digital offerings, DTx require a healthcare professional to prescribe a DTx or diagnose the disease. However, even primary care settings in Germany are considered to have special potential for providing digital services for depressive disorders (64). In contrast to the previous determinants, GPs' influence is a context factor for users' acceptance (65).

Although doctors have been assigned an important role in the prescription of DTx, our results showed that GPs are not perceived as competent counsellors on mental health issues. Especially DTx-interested patients were critical about this specific knowledge in routine care. GPs' influence on the intention to use DTx was limited to a specific group of patients. These results support prior research by Uncovska et al. (2023), which concluded that the physicians' prescription had a minor role in the willingness to use DTx (46). Further, patients in our sample were familiar with various technologies for depressive disorders beyond the healthcare services of routine care and without coordination of their GP. Nevertheless, due to prescription, GPs still have a crucial role for DTx access.

At present, the role of GPs in routine care after a prescription is not very specified. As DTx are considered additionally to in-person care (66, 67), GPs have the potential to provide low-threshold guidance. Thus far, GPs' guidance could compensate for adverse effort and performance expectancy of patients (e.g., less commitment or unreliable use) during bridging waiting times or motivate patients for DTx use. Further guidance on digital interventions is necessary for effective implementation in routine care (10, 68) and could also increase patients' adherence (13). The combination of application-based and in-person care is already practised in psychotherapy, known as blended therapy (17, 29, 69, 70), with a growing interest of patients and therapists (71) and is already discussed as a gold standard for internet and mobile application used for depressive disorders (55).

### 4.3 Strengths and limitations of the study

This study has a few limitations to be noted when interpreting the findings. Our study examines the intention to use DTx, therefore we cannot make any conclusions about the actual use of technologies. Other studies show that the dropout in the actual use of digital intervention in routine care is meaningful (70). Further, a selection bias may have resulted that patients with an affinity for digital technologies tend to be more willing to participate in a video-based interview study. Additionally, interviews were conducted in German and translated, which may affect the tonality.

As a main strength, this study uses an exploration approach for further development of acceptance research, as patients' perspective is not yet widely considered. Thus far technology acceptance research is currently dominated by research on health professionals' perspectives. In terms of access, we reached patients with depressive disorders in their real-world environment. Therefore, the results contribute to understanding factors influencing patients' DTx acceptance within a GP setting.

### 4.4 Implications for clinical practice

Since it is known that patients with positive perceptions towards digital health interventions may benefit more from the offerings (60), it is relevant to consider how patients access DTx in routine care. Further, our results show that the relationship between patient and GP affects DTx acceptance differently. This observation leads to the suggestion that further health professionals should be targeted within the implementation process for digital interventions (72, 73). Therefore, further research should specify how DTx could be integrated into routine care for patients without specialised therapeutical treatment.

## 5 Conclusion

In conclusion, patients identified facilitators and barriers to patients' performance and effort expectancies affecting the intention to use DTx. Also, we identified *health status* and *experience with depressive disorders* as additional determinants of DTx-acceptance. For further development on DTx acceptance, a multidimensional integration of acceptance determinants is needed. Future research should also specify how DTx could be integrated into routine care to strengthen user acceptance in GP primary care.

## Data Availability

The raw data supporting the conclusions of this article will be made available by the authors, without undue reservation.
