# Supplementary material for: A qualitative interview study of patients' attitudes towards and intention to use digital interventions for depressive disorders on prescription
Source: Front Digit Health. 2024 Feb 5;6:1275569. doi: 10.3389/fdgth.2024.1275569 (PMC10875127; doi:10.3389/fdgth.2024.1275569)
Supplement: Supplementary file 1 [file Table1.pdf]

Interviewguide

Translation from German

A) Experience with depression and digital health interventions for depressive disorders

- Please tell me about your current situation: What does it look like? Do you see your GP or other health care provider according to your depression?
- How satisfied are you with the current situation?
    - Are there any specific challenges?
    - What is the role of your GP within this current situation?
- Do you have experience using apps or other digital services related to your condition? Please describe your experience.
    - What were positive/ negative aspects?
    - Please explain what kind of apps/services you used
    - How did you find them?
- Have you heard of "apps on prescription" or digital health applications (DTx)?
    - Tell me about your experiences using digital health applications
    - Do you have experience with similar apps or offerings?

B) Potential chances, risks, and barriers of DTx

- After watching the video: what is your first thought about DTx?
- Are there any advantages from your perspective? Which ones?
- Do you see any disadvantages or risks? Which ones?

C) DTx within a primary medicine setting provided by GPs

- How do you think about your GPs' attitude towards DTx?
- How relevant is his or her opinion to you in this context?

D) Participants' intention to use DTx

- When you think about your own depression, can you imagine any helpful aspects of using DTx?
- How do you rate your own skills in using DTx? (e.g. digital competences, …)
    - What would you expect users need to use a DTx?
- Could you imagine using a DTx? Why would you or why would you not?
    - What must a DTx to contain to be interesting for you?
